# Beneficial Effect of Covalently Grafted α-MSH on Endothelial Release of Inflammatory Mediators for Applications in Implantable Devices

**DOI:** 10.1371/journal.pone.0150706

**Published:** 2016-03-03

**Authors:** Guillaume Le Saux, Laurent Plawinski, Sylvain Nlate, Jean Ripoche, Thierry Buffeteau, Marie-Christine Durrieu

**Affiliations:** 1 Univ. Bordeaux, CBMN, UMR 5248, F-33600, Pessac, France; 2 Univ. Bordeaux, BIOTIS, INSERM U1026, F-33076, Bordeaux, France; 3 Univ. Bordeaux, ISM, UMR 5255, F-33400, Talence, France; Centro Cardiologico Monzino IRCCS, ITALY

## Abstract

Intravascular devices for continuous glucose monitoring are promising tools for the follow up and treatment of diabetic patients. Limiting the inflammatory response to the implanted devices in order to achieve better biocompatibility is a critical challenge. Herein we report on the production and the characterization of gold surfaces covalently derivatized with the peptide α-alpha-melanocyte stimulating hormone (α-MSH), with a quantifiable surface density. *In vitro* study demonstrated that the tethered α-MSH is able to decrease the expression of an inflammatory cytokine produced by endothelial cells.

## Introduction

Diabetes currently affects more than 300 million people around the world and is expected to become the 7^th^ cause of death by 2030 [1]. Patients suffering from diabetes are highly affected by damaging and life-threatening complications [2] and a tighter control of glucose levels is critical to avoid these [3]. To date, the most common method to monitor blood glucose level is the use of portable amperometric sensors with the drawback that it only gives a “snapshot” of the blood glucose levels and necessitates daily repeats of blood sample collection by finger pricking. Continuous glucose monitoring (CGM) could revolutionize the monitoring and management of glycaemia [4, 5]. Today, CGM is performed by quantifying glucose levels within the interstitial fluid, using either non-invasive techniques such as optical and transdermal sensors [6] or invasive subcutaneous sensors [7]. There is, however, a lag between blood and interstitial fluid glucose levels [8] which makes difficult a fast and accurate response to changing glucose concentrations. In the case of subcutaneous sensors, in addition to problems with accuracy [9], biocompatibility is an issue that remains to be fully addressed [10] especially with regards to foreign body reaction [11] which has a negative impact on the subcutaneous sensor’s longevity and performance [11–13]. A less common method is the direct monitoring of blood glucose levels using a closed loop system, consisting of an insulin delivery pump coupled with an intravenous implantable sensor [14]. The intravenous glucose sensor, which was introduced by Armour *et al*. [15], was initially thought to be dangerous because of clotting issues. However, Armour *et al*. showed higher accuracy and encouraging results regarding safety as they delivered real-time blood glucose data with no sign of thrombosis on the animals used in the study.

One issue that remains to be addressed is the role of endothelial cells in the host inflammatory response to the implanted sensor [16]. Endothelial cells are strongly involved in vascular inflammation in part by producing pro-inflammatory cytokines, including interleukin-6 (IL-6), which in turn amplifies leukocyte recruitment [17, 18]. The extent of inflammation is correlated to the expression of cytokines [19] and can eventually lead to a loss in sensor function [20]. To overcome this, a wide range of approaches has been sought to optimize host inflammatory response [21]. Several options are currently in development such as passive anti-inflammatory coatings [22], biocompatible coatings made of natural substances like alginate [23], chitosan [24], collagen [25] or hyaluronan [26] or synthetic hydrogels such as poly(hydroxyethyl methacrylate) [27], poly(ethylene glycol) [28, 29] and poly(lactic-co-glycolic acid) [30]. However, these methods may either trigger an immunogenic response [31] or display poor adhesion and biocompatibility [32]. Hence the need for the development of active anti-inflammatory strategies [33]. Coatings delivering anti-inflammatory agents such as dexamethasone [34, 35] or vitamin E [36] offer an interactive and directed approach to modulate cell behavior, however the efficacy and lifetime of such systems remain limited by poor control over the release kinetics [37] and by the quantity of bioactive molecules they can supply [38].

Using surface immobilized molecules as an alternate strategy could be beneficial for the design of implants with long term anti-inflammatory properties. A candidate with great potential is the alpha-melanocyte stimulating hormone, or α-MSH. This tridecapeptide sequence of the melanocortin family is produced by many different cell types and is known to have potent anti-inflammatory properties [39]. α-MSH exerts its activity via a group of melanocortin receptors (MC-R) belonging to the family of G-protein-coupled receptors [40]. In addition to the central nervous system and melanocytes, MC-R have been detected recently on adipocytes, keratinocytes, immunocompetent as well as inflammatory cells, fibroblasts and importantly, endothelial cells [39, 41, 42].

With regards to electrochemical glucose monitoring, the choice of material is crucial and for this, gold based implants hold great promise [10]. First, gold is the conductive material *par excellence*. Second, gold is widely used in medical devices due to its excellent biocompatibility [43]. Third, gold implants can be ameliorated by functionalizing their surface with bioactive molecules for the control of a desired biological response [44]. Importantly, the bio-interface should be homogeneous, it should have a controlled surface density of active molecules and should avoid undesired interactions with cells [45, 46].

**Fig 1:**
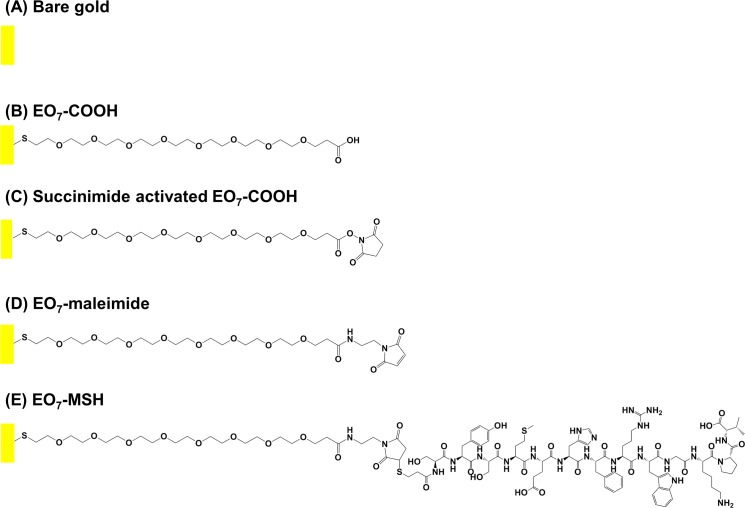
The surfaces used in this study. Schematic depiction of bare gold (A) before and (B) after modification with HS-EO_7_-COOH; “EO_7_-COOH” surface. After (C) activation of the carboxyl moieties with EDC/NHS, (D) the surface is functionalized with maleimide; “EO_7_-maleimide” surface. Finally, (E) α-MSH is immobilized on the surface via thiol-maleimide chemistry; “EO_7_-MSH” surface.

Given the anti-inflammatory action of soluble α-MSH *in vitro* and that it was shown that α-MSH remained active in reducing the inflammatory response of neurons, when immobilized on surfaces [47], we sought to investigate its impact on endothelial behavior. In the present work, we covalently immobilized α-MSH onto gold using well defined self-assembled monolayers of carboxyethyl terminated hepta (ethylene glycol) (see Fig 1 for a depiction of the surfaces used in this study). The produced surfaces were characterized in depth by X-ray Photoelectron Spectroscopy (XPS) and Polarization Modulation Infrared Reflection-Absorption Spectroscopy (PM-IRRAS) to assess their quality and to quantify the effective grafting of α-MSH. We then investigated the impact of the α-MSH modified substrates on lipopolysaccharide (LPS) induced inflammatory response of human umbilical vein endothelial cells.

## Experimental Section

### Materials and chemicals

All chemicals were used as received without further purification. Ultra-pure water (18.2 MΩ.cm; Elga) was used for surface cleaning. *O*-(2-Carboxyethyl)-*O*′-(2-mercaptoethyl)heptaethylene glycol (≥95%; HS-EO_7_-COOH), *N*-(2-Aminoethyl)maleimide trifluoroacetate salt (≥98%), *N-*hydroxysuccinimide (98%; NHS), *N*-(3-Dimethylaminopropyl)-*N*′-ethylcarbodiimide hydrochloride (≥98%; EDC), 2-(N-Morpholino)ethanesulfonic acid hydrate (≥99.5%; MES), absolute ethanol (≥99.8%), methanol (≥99.6%) and sulfuric acid (85.0–98.0%) were purchased from Sigma-Aldrich, France. Hydrogen peroxide (35% w/w in water) was purchased from Alfa Aesar, Germany. Custom synthesized HS-CH_2_-CH_2_-CO-Ser-Tyr-Ser-Met-Glu-His-Phe-Arg-Trp-Gly-Lys-Pro-Val-NH_2_ peptide (≥98%; α-MSH) was obtained from Genecust, Luxemburg. Gold coated glass slides (300 nm thickness evaporated gold on glass-plates with an intermediate layer of Cr/Ni) were purchased from Applications Couches Minces, Paris.

### Preparation of SAMs on gold

The successive modification steps and resulting surfaces are schematically displayed in Fig 1. The formation of *O*-(2-Carboxyethyl)-*O*′-(2-mercaptoethyl)heptaethylene glycol derived self-assembled monolayers (SAMs) was performed according to the protocol provided by Sigma-Aldrich [48]. Gold plated glass slides were cut into 1cm^2^ pieces for XPS and cell experiments or 4 cm^2^ for PM-IRRAS experiment and cleaned for 30 min in freshly prepared piranha solution (1:3 v/v H_2_O_2_ to H_2_SO_4_). After copious rinsing with, consecutively, ultra-pure water and absolute ethanol, the surfaces were immersed in a 20 mM solution of HS-EO_7_-COOH in absolute ethanol for 24 h. Subsequently, the samples were rinsed with absolute ethanol, sonicated for 2 min in fresh ethanol and dried under a stream of nitrogen. The produced surfaces are henceforth referred to as EO_7_-COOH (surface B in Fig 1).

### Functionalization of gold surfaces with α-MSH

The EO_7_-COOH surfaces were immersed for 1 h in an aqueous solution of EDC, NHS and MES at concentrations of 0.2, 0.1, and 0.1 M respectively thus forming the succinimide activated EO_7_-COOH (Fig 1C). After copious rinsing with consecutively ultra-pure water and methanol, the surfaces were immersed for 12 h in a 10 mM methanolic solution of *N*-(2-Aminoethyl)maleimide trifluoroacetate salt to give EO_7_-maleimide (Fig 1D). The surfaces were then rinsed extensively with methanol, ethanol and water and immersed for 2 h in a 1 mM aqueous solution of the thiolated α-MSH (EO_7_-MSH surfaces, Fig 1E). After covalent immobilization, the surfaces were rinsed with ultra-pure water for 1 week under agitation in order to remove the physically adsorbed peptides.

### Surface characterization

#### X-ray Photoelectron spectroscopy (XPS)

A ThermoFisher Scientific K-ALPHA spectrometer was used for surface analysis with a monochromatized AlKα source (hν = 1486.6 eV) and a 200 micron spot size. A pressure of 10^−7^ Pa was maintained in the chamber during analysis. The survey spectra (0–1350eV) were obtained with a constant pass energy of 200 eV and high resolution spectra at a constant pass energy of 40eV. Charge neutralization was activated even for conductive samples. High resolution spectra were fitted and quantified using the AVANTAGE software provided by ThermoFisher Scientific. Two samples per condition were prepared and three areas per sample were analyzed.

#### Polarization modulation-infrared reflection-adsorption spectroscopy (PM-IRRAS)

PM-IRRAS spectra were recorded on a ThermoNicolet Nexus 670 FTIR spectrometer at a resolution of 4 cm^-1^, by coadding several blocks of 1500 scans (30 minutes acquisition time). All spectra were collected in a dry-air atmosphere after 30 min of incubation in the chamber. Experiments were performed at an incidence angle of 75° using an external homemade goniometer reflection attachment [49]. The infrared parallel beam was directed out of the spectrometer with an optional flipper mirror and made slightly convergent with a first BaF_2_ lens. The IR beam passed through a BaF_2_ wire grid polarizer (Specac) to select the p-polarized radiation and a ZnSe photoelastic modulator (PEM, Hinds Instruments, type III) which modulates the polarization of the beam at a high fixed frequency (74 KHz) between the parallel (p) and perpendicular (s) linear states. After reflection on the sample, the double modulated (in intensity and in polarization) infrared beam was focused with a second ZnSe lens onto a photovoltaic MCT detector (Kolmar Technologies, Model KV104) cooled at 77 K. In all experiments, the PEM was adjusted for a maximum efficiency at 2500 cm^-1^ to cover the mid-IR range in only one spectrum. For calibration measurements, a second linear polarizer (oriented parallel or perpendicular to the first preceding the PEM) was inserted between the sample and the second ZnSe lens. This procedure was used to calibrate and convert the PM-IRRAS signal in terms of the IRRAS signal (i.e., $1-\frac{R_{p}\left(d\right)}{R_{p}\left(0\right)}$ where *R*_*p*_(*d*) and *R*_*p*_(0) stand for the p-polarized reflectance of the film/substrate and bare substrate systems, respectively) [50, 51].Two samples per condition were prepared for PM-IRRAS analysis.

### Cell studies

#### Cell culture

Human Umbilical Vein Endothelial Cells (HUVEC) were purchased from PromoCell, France and were cultured in PromoCell’s Endothelial Cell growth medium-2 at 37°C in 5% CO_2_. For experiments, cells were used between passages 4 and 6.

#### Cell Adhesion

In a 24-well plate (Falcon Multiwell, Becton Dickinson & Co., NJ., USA), 500 μL of HUVEC suspension were incubated on the 1 cm^2^ surfaces at a density of 100000 cells per mL in serum free EBM-2 (Lonza, Switzerland) medium for 4–6 h. After this, the medium was replaced with EGM-2 BulletKit medium (Lonza, Switzerland) and cells were left to adhere for 24 h on the different surfaces used in this study. As a control, cells were also seeded cells on the plastic of the 24-well plate. To assess the specific effect of surface bound molecules on endothelial adhesion and inflammatory response, the Bulletkit aliquot which contained hydrocortisone was not added to the EGM-2 culture medium.

#### Fluorescence microscopy

Immunofluorescent staining was performed to visualize the HUVECs on the different surfaces. After cell culture, cells were fixed by 4% (v/v) paraformaldehyde, permeabilized with 0.5% Triton-X 100, blocked with 1% bovine serum albumin (BSA) in PBS solution. The actin cytoskeleton was stained with Alexa Fluor® 488 phalloidin (Invitrogen, France). Nuclei were stained by mounting the samples with ProLong® Gold antifade reagent containing DAPI (Invitrogen, France). Cell adhesion was imaged using a Leica DM5500B epifluorescence microscope and quantified using the ImageJ software (NIH, http://rsb.info.nih.gov/ij/). Cell nuclei were counted for evaluation of adherent cell numbers. At least 10 fields at low magnification (10 X) on each surface were analyzed for this study.

#### IL-6 production

HUVECs were replated onto the surfaces at a density of 50000 cells /cm^2^ according to the protocol stated previously. HUVEC were stimulated by adding 1 μg/mL of lipopolysaccharide (LPS, from E. Coli 0111:B4; Sigma-Aldrich, France) at the moment of replating. Negative control was performed by incubating cells in regular culture medium. IL-6 concentrations in the culture media of treated cells were measured using the human Instant IL-6 ELISA kit (eBioscience, Austria). IL-6 production was normalized to the number of adherent cells which was obtained as described above. All procedures were performed according to the manufacturer’s instructions. IL-6 levels where measured using an ELx808 microplate reader at 450 nm (Biotek, France).

#### Statistics

All samples were assayed in triplicate and experiments were repeated 5 times. Statistical analysis was performed by analysis of variance. A Bonferroni multiple-comparison post-hoc test was also performed using the Prism software (GraphPad Software Inc., USA). P-values are reported for comparisons of interest.

## Results

### Functionalization of the surfaces

#### XPS analysis

Table 1 gives the changes in atomic proportions of surfaces used in this study.

**Table 1:** Experimental atomic composition (%) obtained by XPS.

Atomic %	Au	S	C	O	N	Impurities	C/N	C/O
Bare Gold^a^	35.9	<1	42.9	16.4	—	4.1	—	2.61
EO_7_-COOH^a^	42.4	1.7	38.5	17.4	—	—	—	2.21
EO_7_-maleimide	49.6	3.6	31.7	12.6	2.5	—	12.68	2.52
EO_7_-MSH	30.9	1.9	44.5	18.0	4.7	—	9.47	2.4

^a^See S1 Text.

**Fig 2:**
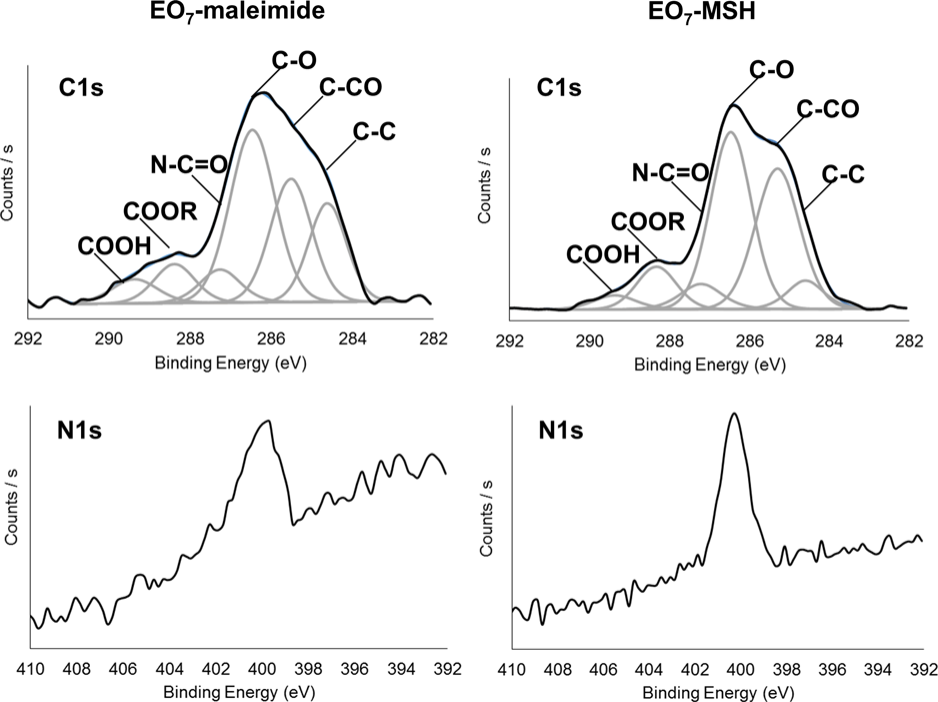
XPS analysis of maleimide linker and peptide modified surfaces. C1s and N1s high resolution spectra of the EO_7_-maleimide and EO_7_-MSH surfaces.

A detailed analysis confirming the effective modification of bare gold with HS-EO_7_-COOH to obtain the EO_7_-COOH surfaces is described in S1 Text. The subsequent modification steps are shown in Fig 2.

To prevent any hydrolysis of the succinimidyl ester (Fig 1C) which would have lowered subsequent coupling yields, we proceeded directly to the maleimide and α-MSH modified surfaces. The C1s and N1s spectra of EO_7_-maleimide surface is shown on the left in Fig 2. In addition to the C-C at 284.6 eV, C-O at 286.3 and COOH carbons at 289.3, we observe the presence of new carbon species such as the C-CO at 285.3 eV, COOR at 288.4 eV and importantly the N-C = O carbons at 287.4 eV which are characteristic of expected chemical groups (Fig 1D). The N1s spectrum exhibits a contribution centered at 400 eV which corroborates the presence of N-C = O carbons. The experimental C/O and C/N ratios are 2.52 and 12.68, respectively (Table 1) which are close to the theoretical ratios of 2.27 and 12.5 in the case of a fully modified surface. The C1s and N1s spectra of the α-MSH modified surface are displayed in Fig 2 (right). In addition to the contributions of C-C at 284.8 eV, C-CO at 285.7 eV, C-O at 286.6 eV, COOR at 288.6 eV and COOH at 289.5 eV carbons, we observe the presence N-C = O at 287.6 eV as well as an increase in nitrogen from 2.5 to 4.7% (Table 1) which confirms the presence of peptides on the surface. For the EO_7_-MSH surface (Fig 1E), we found C/N and C/O ratios of 9.47 and 2.4 respectively (Table 1). These are significantly different to the theoretical ratios of 4.68 and 3.32 for a fully modified surface and thus suggest an incomplete modification of the surface. To assess the coupling efficiency, we took a closer look at the C/N ratios of theoretical and experimental surfaces. For a 100% percent coupling efficiency, or a surface fully modified with α-MSH, the expected C/N ratio is equal to 4.68. In contrast, a 0% coupling equates to having an EO_7_-maleimide only surface. We showed previously that the corresponding C/N was 12.68 experimentally. In the present case, a C/N ratio of 9.47 equates to a 40.1% coupling efficiency. We then used PM-IRRAS to confirm coupling yields and assess peptide surface densities.

#### PM-IRRAS analysis

**Fig 3:**
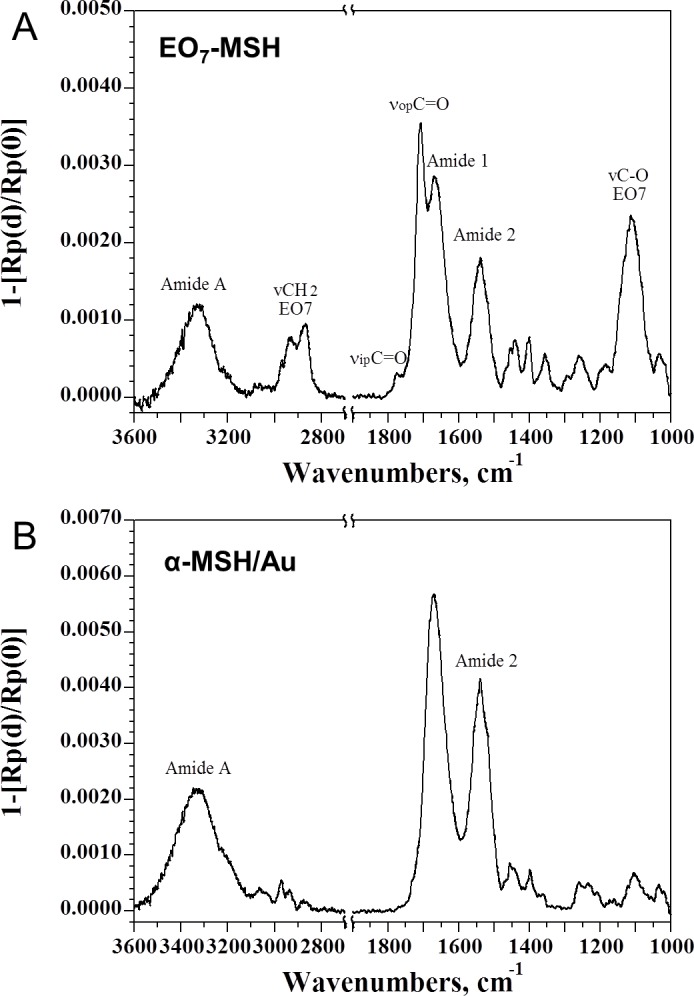
PM-IRRAS analysis. Spectra, expressed in IRRAS units, of (A) EO_7_-MSH surface and (B) compact monolayer of α-MSH.

The EO_7_-COOH was also analyzed by PM-IRRAS (see S1 Text), and showed that the surface is characteristic of crystalline PEO possessing a (7/2) helix structure with a succession of trans, trans and gauche conformations of the seven ethylene oxide units which turn two times per fiber period [52]. Its symmetry properties are described by the D_7_ point group, allowing two active symmetry classes, in infrared spectroscopy, for each vibrational mode: A_2_ with the transition moment along the helix axis and E_1_ with the transition moment normal to the helix axis [53]. The ethylene oxide helices are therefore oriented along an axis normal to the gold surface. The peptide modified surface EO_7_-MSH was also analyzed by PM-IRRAS (Fig 3A).

Characteristic bands of the seven ethylene oxide (EO_7_) units are observed, such as at 2891 cm^-1^, corresponding to the C-H stretching of CH_2_ and at 1117 cm^-1^ assigned to the symmetric C-O-C stretching vibration. These bands are broader than for the crystalline PEO [52] and suggest a slight loss if EO_7_ backbone’s crystalline nature upon peptide grafting. The presence of the maleimide linker is confirmed by the two ν_ip_C = O and ν_op_C = O bands at 1760 and 1700 cm^-1^, respectively. We also observe the appearance of amide bands at 1663 and 1549 cm^-1^ which account respectively for the amide I (νC = O) and amide II (δCNH + νC-N) modes of α-MSH. To assess the coupling efficiency, we compared the intensity of the amide bands from the PM-IRRAS spectrum of the EO_7_-MSH surface with that of a monolayer of our thiolated α-MSH that was directly deposited on gold (Fig 3B). The coupling yield of α-MSH the EO_7_-maleimide surface was found to be 50.1% which is substantially higher than the 40.1% found by XPS. Even though the underlying EO_7_-COOH surface’s near crystallinity is partially disrupted, it is possible to quantify the maximal surface density. Takahashi *et al*. described in detail the crystal structure of PEO, they observed that four helices pass through the PEO unit cell with parameters a = 8.05 Å, b = 13.04 Å, c (helix axis) = 19.48 Å and β = 125.4° [52]. Assuming that the freestanding carboxyl units do not disrupt the PEO backbone, we can therefore calculate the number of available carboxyl molecules per mm^2^ (see S2 Text for calculation details). Ensues the amount of covalently grafted peptides per mm^2^ and thus the surface density which derives from the coupling yields obtained from XPS and PM-IRRAS. The surface density was found at 3.5 ± 0.5 pmol per mm^2^ for the EO_7_-MSH substrate.

### Comparison of immobilized α-MSH versus soluble α-MSH

**Fig 4:**
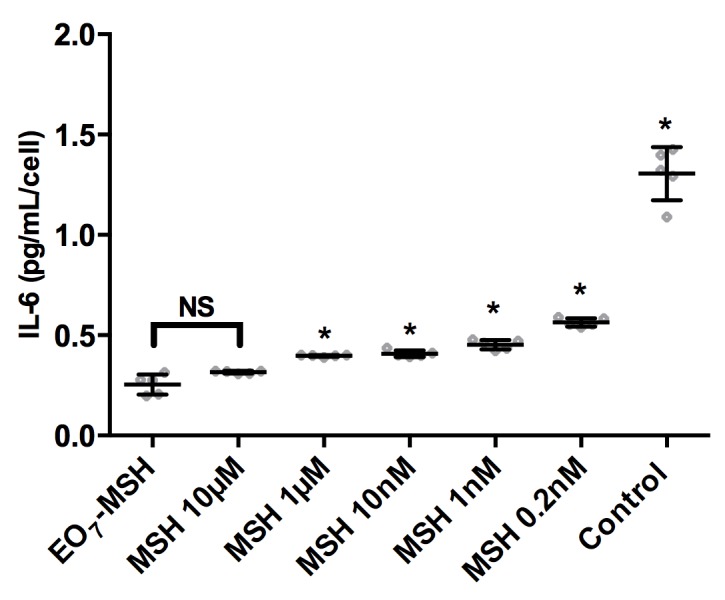
Soluble *versus* surface bound α-MSH. Effect of immobilized α-MSH (EO_7_-MHS) on LPS induced endothelial IL-6 production compared to cells re-plated onto culture dishes in culture medium containing either no soluble α-MSH (Control) or soluble α-MSH in concentration ranging from 0.2 nM to 10 μM. Error bars represent standard deviations. A Bonferroni test relative to EO_7_-MSH vs. other treatments was performed. Differences were considered significant for p<0.01 and marked with an asterisk. The number of replicates is n = 5.

We investigated the potency of surface bound α-MSH compared to its soluble form. For this, it was imperative to quantify the total number of attached molecules per surface and to compare their efficacy with the same amount of molecules in solution. Knowing the surface density of α-MSH by PM-IRRAS, we could quantify the total number of available molecules for a given surface area. Considering a density of 3.5 pmol/mm^2^, the total number of MSH molecules for a 1 cm^2^ substrate is 350 pmoles. Given that 500 μL of cell medium is used per well for a 24 well plate, the equivalent number of soluble α-MSH is found for a concentration of 0.7 μM. The anti-inflammatory effect of soluble α-MSH was observed *in vitro* at concentrations from 1 nM to 1 μM [54]. Therefore, we prepared cell culture media containing α-MSH with concentrations ranging from 0.2 nM to 10 μM, replated cells on plastic according to the protocol stated previously, stimulated cells with 1 μg/mL LPS and quantified IL-6 production by ELISA. Results are shown in Fig 4.

As expected, the presence of α-MSH for concentrations as low as 0.2 nM significantly decreases IL-6 expression as shown by analysis of variance (p < .0001). We then compared EO_7_-MSH to the other treatments and found that, with the exception of MSH 10 μM condition, IL-6 production was significantly lower on the EO_7_-MSH (p<0.01).

### Impact of the EO_7_-MSH surface on HUVEC IL-6 production

**Fig 5:**
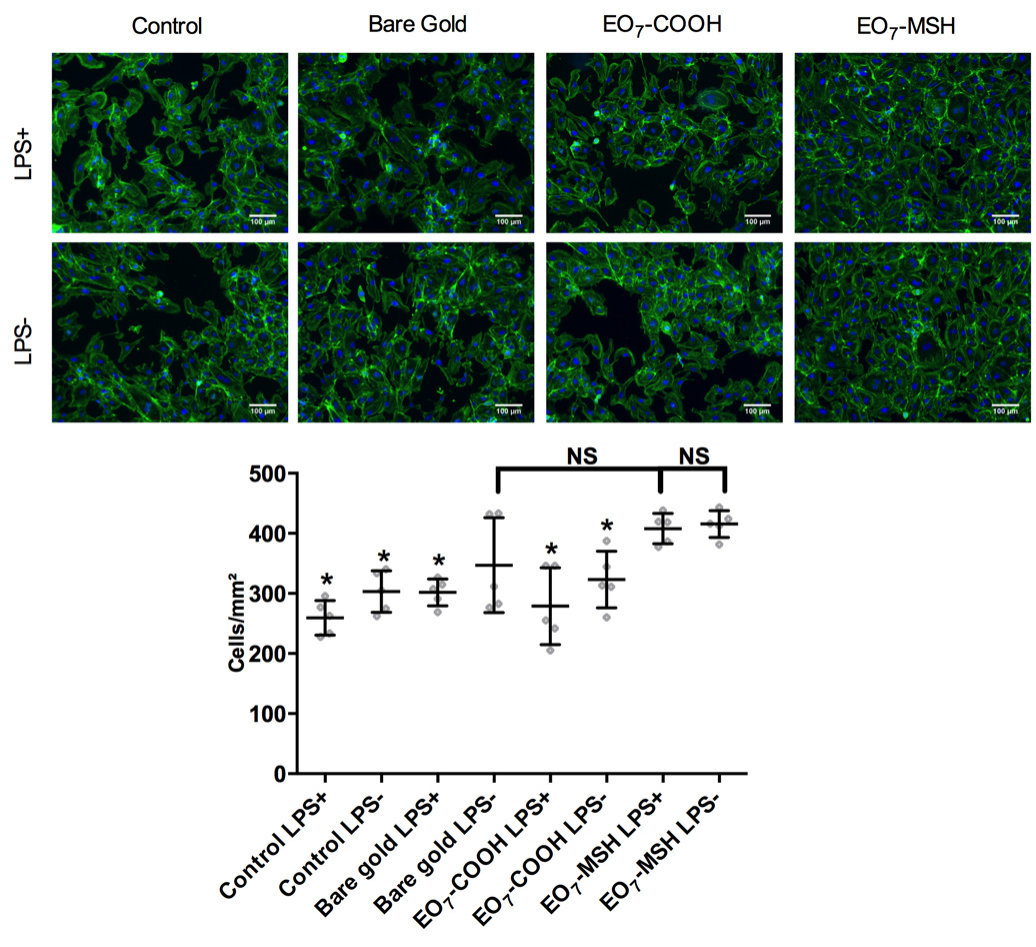
HUVEC adhesion. (A) Epifluorescence images of Endothelial cells that were re-plated onto bare gold, EO_7_-COOH, EO_7_-MSH surfaces and culture dish (control) in culture medium containing 1μg/mL lipopolysaccharide (LPS+) or normal culture medium (LPS-). Cell were stained for actin (green) and nuclei (blue). (B) The fluorescence images were analyzed to determine the average number of adherent cells per mm^2^ after cells were adhered for 24h. Error bars represent standard deviations. A Bonferroni test relative to EO_7_-MSH LPS+ vs. other surfaces was performed. Differences were considered significant for p<0.01 and marked with an asterisk. The number of replicates is n = 5.

Prior to assessing HUVEC inflammatory response on the modified gold, cell adhesion was quantified on the surfaces used in this study (Fig 5).

**Fig 6:**
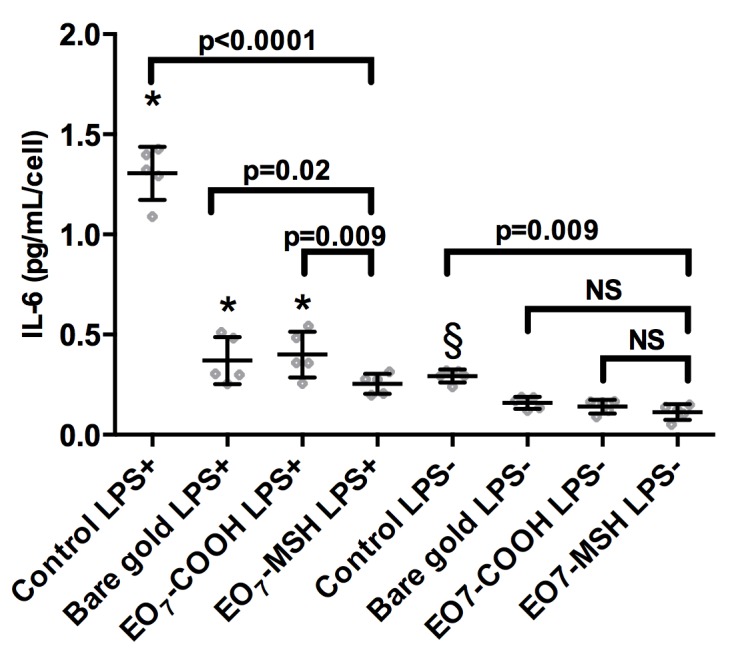
Surface bound α-MSH *versus* other surfaces. IL-6 production of endothelial cells that were re-plated onto bare gold, EO_7_-COOH, EO_7_-MSH surfaces and culture dish (control) in culture medium containing 1μg/mL lipopolysaccharide (LPS+) or normal culture medium (LPS-). Error bars represent standard deviations. A Bonferroni test relative to EO_7_-MSH LPS+ vs. other LPS+ surfaces was performed. Differences were considered significant for p<0.05 and marked with an asterisk. Another post-hoc test comparing EO7-MSH LPS- to other LPS- surfaces was performed. Differences were considered significant for p<0.05 and marked with a section sign. The number of replicates is n = 5.

As seen in Fig 5A, HUVECs adhere on the surfaces and have a well-organized actin cytoskeleton. Overall, treatment with LPS does not appear to induce a change in cell morphology when compared to normal conditions. Cell adhesion per unit area is shown in Fig 5B. Analysis of variance showed significant differences in adhesion (p<0.003). Bonferroni post-hoc testing showed that, save for Bare gold LPS-, cell adhesion was highest for EO_7_-MSH surfaces, regardless of LPS treatment (p<0.01). Importantly, we observe no significant differences in adhesion on EO_7_-MSH surfaces between normal condition and LPS treated cell (p>0.99). We next investigated the effect of α-MSH grafted to surface on HUVEC (Fig 6).

LPS, which is known to stimulate endothelial cell production of IL-6 [55], induced as expected a four-fold increase in IL-6 production by HUVECs in our controls on plastic culture dishes. Analysis of variance showed significant differences in IL-6 production (p<0.0001). Overall, IL-6 production was lower on all gold surfaces than on plastic culture dishes. Post-hoc analysis by Bonferroni showed that although IL-6 production is lower on EO_7_-MSH than on plastic for unstimulated cells (p<0.0001), the EO_7_-MSH surface did not fare significantly better than the bare gold or EO_7_-COOH surfaces (p>0.99). Conversely, in LPS stimulated cells, the lowest production of IL-6 is observed for the EO_7_-MSH surface when compared to the positive control (p<0.0001), the bare gold (p = 0.02), and EO_7_-COOH surface (p = 0.009) thus indicating anti-inflammatory activity of surface grafted α-MSH on HUVECs in pro-inflammatory conditions.

## Discussion

Gold substrates modified with bioactive molecules have been widely used to study cell behavior [45, 46]. It is generally assumed that the amount of peptide is proportional to both the gold surface area and to the bulk concentration. However, evaluating the surface density of active principles remains arduous. Numerous reports confirm the effective modification of the gold substrate with peptides but little to no information is given on the quantitative evaluation of surface density [56–58]. In contrast, when quantifying surface density is possible using STM on atomically smooth gold, it is difficult to implement this type of substrate for biological experiments [59]. Others were able to quantify the interparticle distance of large dendrimers on gold by AFM [60] but since RGD clustering is known to strongly impact cell adhesion[61], the technique described by Lagunas *et al*. does not allow to investigate the impact of a known homogeneous peptide distribution on cell behavior. Using XPS and PM-IRRAS, we confirmed the effective grafting of α-MSH onto gold via a carboxyl terminated oligo(ethylene oxide) SAM. Serendipitously, we found that this SAM had the crystal structure of poly(ethylene oxide). In conjunction with the coupling yield obtained by XPS and PM-IRRAS, we therefore found a facile method to quantify the surface density of α-MSH which was found to be 3.5 pmol/mm^2^. This result is not only important because it distinguishes itself from the previously mentioned works where peptide density was only estimated but also because we were able to correlate this density with the activity of α-MSH either surface bound or in solution.

Since other molecules possess notable anti-inflammatory properties when grafted onto surfaces, it is of interest to compare their relative potency. For example, an *in vivo* study demonstrated the potency of superoxide dismutase in reducing foreign body reaction [62]. However, in this case, surface density was only indirectly quantified, making a comparison impossible. In another case, catheters were modified with heparin with densities of 10 to 100 fmol/mm^2^ to block the adsorption of pro-coagulant proteins[63]. Kim *et al*. studied the effect of covalently immobilized rhIL-1ra-ELP fusion protein on the inflammatory response of LPS-stimulated human monocytes[64]. They showed that a surface density of 35 fmol/mm^2^ inhibited monocyte IL-6 production by 50%. Considering that, in our case, a surface density of 3.5 pmol/mm^2^ of α-MSH reduced by 80% the production of IL-6 by LPS-stimulated HUVEC it appears that surface bound α-MSH fares at least as well as other anti-inflammatory molecules such as heparin or IL-1. However, because surface bound α-MSH with a density of 0.21 pmol/mm^2^, or 16 times less than in our case, reduced IL-1 and TNFα production by 2 to 3 orders of magnitude in microglia [47] it cannot be excluded that the potency of a given molecule is not uniform for all cell types.

To our knowledge, the impact of gold surfaces on endothelial IL-6 production *in vitro* is yet to be investigated. However, in an *in vivo* model, others showed that the nature of the surface modification elicited different level of inflammatory response, with bare gold inducing the lowest levels of inflammation [65]. Our study did indeed show less IL-6 production by HUVEC plated on bare gold. In addition, Tanigawa *et al*. concluded that gold was a useful intravascular material as it reacts minimally with the vessel wall and induces lower thrombogenicity [66]. It must be added that for cardiovascular applications, gold-coated stents do not decrease in-stent restenosis [67]. Restenosis is the re-narrowing of the vessel consecutive to the placement of a stent in an artery and is known to be linked with inflammation [68] and an increase in IL-6 production [69]. For the latter, the authors acknowledged that this increase in IL-6 production could stem from many different cell types and the sole contribution of endothelial cells was not quantified.

It is known that α-MSH in solution, in concentrations ranging from 10^−8^ to 10^−12^ M, has an anti-inflammatory effect on endothelial cells [40, 70]. This decrease in inflammation is done via the NF-κβ pathway in a dose dependent manner [71], and is observed by a reduction in the production of adhesion molecules [72] and pro-inflammatory cytokines [73]. To be active, α-MSH specifically binds the MC-1 receptor which is present on the outer cell membrane [74]. Therefore, and contrarily to glucocorticoids [75], α-MSH does not require internalization to exert its anti-inflammatory action. The grafting of MSH peptides onto gold surface without any loss of bioactivity was successfully achieved. As previously shown, binding biomolecules, such as peptides, directly to the biomaterial surface can result in steric hindrance, conformation change and loss of bioactivity [76]. In this work, it was suggested that the nature of the spacer on top of which is attached the bioactive molecule is decisive in preserving bioactivity. Interestingly, poly(ethylene oxide), which constitutes the base layer in this work, could provide a relatively hydrophilic environment which is helpful in maintaining the bioactivity of certain molecules [77]. This could explain why, in our case, α-MSH remains active *in vitro*. Accordingly, we observed that thiolated α-MSH directly grafted onto gold is not as efficient as EO_7_-MSH in reducing HUVEC IL-6 production (Fig A in S3 Text). However, it must be added that most of the work on α-MSH was previously performed *in vivo* and it was therefore important to investigate how culture media that contained soluble α-MSH in this range affected endothelial IL-6 production or not. As seen here, soluble α-MSH does decrease IL-6 production of endothelial cells by 40 to 80%, depending on the concentration. As a means of comparison, micromolar concentrations of dexamethasone, which is known for its anti-inflammatory properties[78], inhibited IL-6 production of LPS-stimulated HUVEC by 40–50%[79]. This confirms that immobilized α-MSH is at least as efficient in reducing IL-6 production as its soluble form. Considering that longevity is an issue with an α-MSH releasing device [38], future *in vivo* testing of the efficacy of surface immobilized α-MSH is vital for applications where the long term control of endothelial inflammatory response is required.

## Conclusion

In summary, we successfully modified and characterized gold surfaces with α-MSH. Due to the crystalline nature of the base layer, we were able to assess the peptide surface density quantitatively. We then showed that these surfaces can significantly decrease endothelial IL-6 production in response to LPS stimulation. Particular attention should be paid to the surface density of these molecules however. Indeed, as seen here, the effect of α-MSH is dose dependent and decreasing surface density could potentially elicit a less clear effect on endothelial cells. Further work on the impact of density as well as other cell responses to α-MSH and *in vivo* testing are required before direct applications in intravascular glucose sensors can be envisaged but also for subcutaneous sensors [10] and cardiovascular applications [67] which also require perennial anti-inflammatory coatings.

## Supporting Information

S1 Text XPS and PM-IRRAS analyses of the EO_7_-COOH surfaces. (DOCX)

S2 Text Determination of EO_7_-MSH surface density. (DOCX)

S3 Text Impact of thiolated α-MSH on HUVEC IL-6 production. (DOCX)
