# Supplementary material for: Beneficial Effect of Covalently Grafted α-MSH on Endothelial Release of Inflammatory Mediators for Applications in Implantable Devices
Source: PLoS One. 2016 Mar 3;11(3):e0150706. doi: 10.1371/journal.pone.0150706 (PMC4777356; doi:10.1371/journal.pone.0150706)
Supplement: S1 Text — (DOCX) [file pone.0150706.s001.docx]

XPS analysis – The C1s spectrum of bare gold is shown in Fig. A (left).


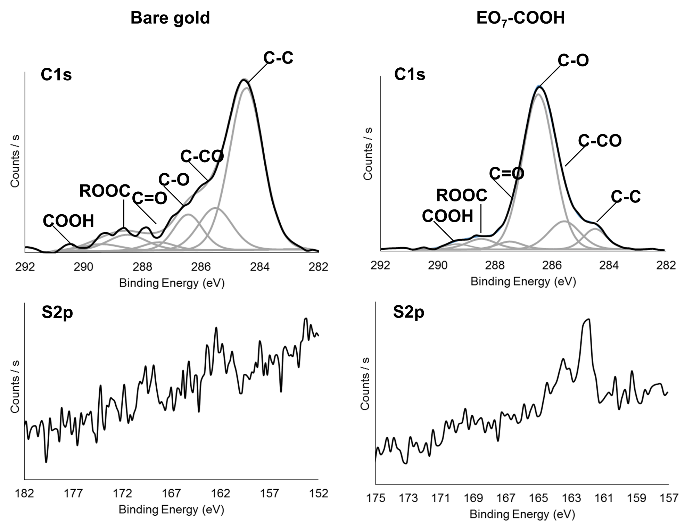


Fig. A. XPS analysis EO_7_-COOH surfaces. C1S and S2p regions of bare gold before modification and after functionalization with HS-EO_7_-COOH.

The surface exhibits adventitious species, mainly aliphatic carbons at 284.5 eV but also various oxidized forms of carbon such as C-CO at 285.5 eV, C-O at 286.4 eV, C=O at 287.4 eV, COOR at 288.5 eV and COOH at 289.4 eV. In addition, traces of sulfur species are observed on bare gold as shown in the S2p spectrum in Fig. S1. All of these contributions stem from atmospheric pollution and are commonly witnessed by XPS on gold. The C1s and S2p spectra of the surface after modification with the carboxyethyl mercapto oligo(ethylene glycol) are shown in Fig. S1 (right). The C1s high resolution scan shows a main peak at 286.3 eV assigned to C-O linked carbons, which stem from the ethylene oxide moieties that constitute the SAM backbone. In addition, the C-CO at 285.6 eV, C=O at 287.5 eV, the COOR at 288.5 eV and the COOH carbons at 289.3 eV suggest an effective modification of the surface with the HS-EO_7_-COOH molecules. Furthermore, the inflexion at 284.5 eV pertaining to C-C species suggests a low adventitious contamination of the surface. The binding energy of the S2p_2/3_ peak is at 162.2 eV is in good agreement with chemisorbed thiols on gold surfaces[[1](#_ENREF_1)]. For the EO_7_-COOH surface, the C/O ratio is equal to 2.21, this value is higher than the theoretical value of 1.72. This is mainly due to the presence of surface contaminants as seen by the contribution at 284.5 eV.

PM-IRRAS analysis – The PM-IRRAS spectrum of the EO_7_-COOH surfaces, expressed in terms of IRRAS units, is shown in Fig. B.


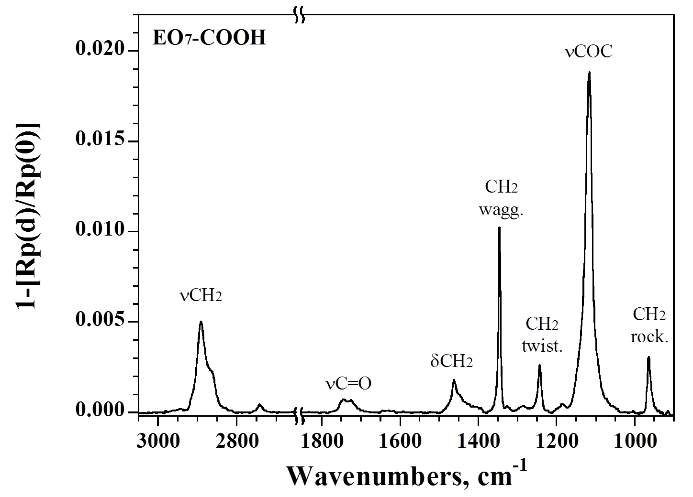


Fig. B. PM-IRRAS spectrum, expressed in IRRAS units, of EO_7_-COOH surfaces.

This PM-IRRAS spectrum exhibits all the characteristic bands of the poly(ethylene oxide) (PEO) moiety [[2](#_ENREF_2)]. Thus, the sharp bands observed at 2891, 1463, 1346, 1243, and 965 cm^-1^ are assigned to the stretching, bending, wagging, twisting and rocking vibrations of the methylene groups, respectively, whereas the band at 1117 cm^-1^ is assigned to the symmetric C-O-C stretching vibration. The wavenumbers and the relative intensity of these bands are very similar to those obtained by Buffeteau et al. [[2](#_ENREF_2)] for a 100 Å thick poly(ethylene oxide) (PEO) films deposited onto aluminum substrates by electrochemistry. This spectrum is characteristic of crystalline PEO possessing a (7/2) helix structure with a succession of trans, trans and gauche conformations of the seven ethylene oxide units which turn two times per fiber period [[3](#_ENREF_3)]. Its symmetry properties are described by the D_7_ point group, allowing two active symmetry classes, in infrared spectroscopy, for each vibrational mode: A_2_ with the transition moment along the helix axis and E_1_ with the transition moment normal to the helix axis [[4](#_ENREF_4)]. The wavenumbers of the stretching, bending, wagging, twisting and rocking vibrations of the methylene groups, observed in our PM-IRRAS spectrum, reveals only the A_2_ component for each mode. Considering the surface selection rule of the PM-IRRAS spectroscopy (i.e. surface absorptions with a transition moment parallel to the substrate are not detected by PM-IRRAS), we can conclude that the ethylene oxide helices are oriented along an axis normal to the gold surface. Furthermore, the terminal carboxylic acid functions give rise to the two bands observed at 1745 and 1724 cm^-1^, ascribed to the stretching vibrations of the carbonyl groups [[5](#_ENREF_5)]. The observation of these two bands indicates the presence of free (1745 cm^-1^) and hydrogen bonded (1724 cm^-1^) carbonyl groups at the surface, revealing the higher degree of freedom for this terminal group. The presence of hydrogen bonded carbonyl groups at the surface comes from the interaction between adjacent carboxylic acid functions in the monolayer. Finally, the intensity (in IRRAS units) around 0.001 for the stretching vibrations of the carbonyl groups is in good agreement with other PM-IRRAS results obtained for SAMs with terminal carboxylic acid functions [[6](#_ENREF_6)].

References

1. Yang YW, Fan LJ. High-Resolution XPS Study of Decanethiol on Au(111): Sulfur-Gold Bonding Interaction. Langmuir. 2002;18(4):1157-64.

2. Buffeteau T, Desbat B, Turlet J-M. FTIR spectroscopy of monolayers and ultrathin films using polarization modulation. Microchimica Acta. 1988;95(1-6):23-6.

3. Takahashi Y, Tadokoro H. Structural Studies of Polyethers, (-(CH2)m-O-)n. X. Crystal Structure of Poly(ethylene oxide). Macromolecules. 1973;6(5):672-5.

4. Siesler HW, Holland-Moritz K. Infrared and raman spectroscopy of polymers. Marcel Dekker, New York. 1980.

5. Ramin MA, Le Bourdon G, Heuzé K, Degueil M, Belin C, Buffeteau T, et al. Functionalized Hydrogen-Bonding Self-Assembled Monolayers Grafted onto SiO2 Substrates. Langmuir. 2012;28(51):17672-80.

6. Ramin MA, Le Bourdon G, Daugey N, Bennetau B, Vellutini L, Buffeteau T. PM-IRRAS Investigation of Self-Assembled Monolayers Grafted onto SiO2/Au Substrates. Langmuir. 2011;27(10):6076-84.
