# Supplementary material for: Beneficial Effect of Covalently Grafted α-MSH on Endothelial Release of Inflammatory Mediators for Applications in Implantable Devices
Source: PLoS One. 2016 Mar 3;11(3):e0150706. doi: 10.1371/journal.pone.0150706 (PMC4777356; doi:10.1371/journal.pone.0150706)
Supplement: S2 Text — (DOCX) [file pone.0150706.s002.docx]

Using XPS and PM-IRRAS, it was found that α-MSH was grafted onto the carboxyl terminated PEO backbone with coupling yields of 40.1% and 50.1% respectively. In addition, PM-IRRAS analysis of the carboxyl terminated PEO self-assembled monolayer (EO7-COOH), revealed the crystalline nature of the PEO backbone. From the work of Takahashi et al. [[1](#_ENREF_1)], its lattice parameters are known (see Fig. S3 for a scheme of the crystal structure of PEO)


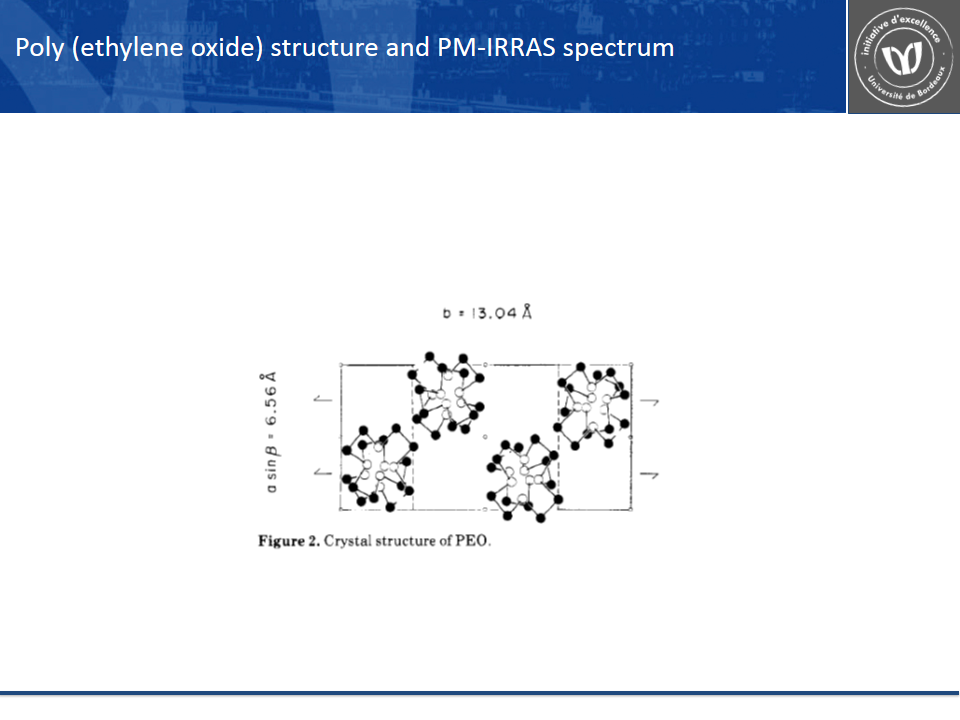


Fig. A. Crystal structure of PEO, courtesy of Takahashi et al.

Knowing the parameters and considering a rectangular lattice as shown in fig. A, we then sought to calculate the number of free-standing carboxyl moieties per mm². Table A, summarizes the values for the lattice parameters as well as the formula to obtain the number of free-standing carboxyl per mm². For ease of use, Takahashi’s number were converted to meters.

Table A. Calculation of the number of free-standing carboxyl moieties per mm²

| Lattice parameter of PEO crystal structure^a^ |  | Formula |
| --- | --- | --- |
| a (in meters) | 8.1E^-10^ |  |
| b (in meters) | 1.3E^-09^ |  |
| β (in degrees) | 125.4 |  |
| aSinβ (in meters) | 6.6E^-10^ |  |
| A (rectangular Lattice area in m²) | 8.6E^-19^ | aSinβ*b |
| 1mm² in m² | 1.0E^-06^ |  |
| N_COOH_ (Nb carboxyl moieties per mm²)^b^ | 4.7E^+12^ | (1.0E^-06^/A)*4 |

^a^Obtained from Takahashi et al.

^b^There are 4 free-standing carboxyl moieties per rectangular lattice

Using the coupling yield obtained from XPS and PM-IRRAS and assuming that a 50% yield equates to 1 carboxyl in 2 being functionalized with α-MSH, we can then calculate the surface density of α-MSH. The results are displayed in Table B.

Table B. Calculation of the surface density of α-MSH

|  | PM-IRRAS | XPS | Formula |
| --- | --- | --- | --- |
| C_Y_ (α-MSH coupling yield in %) | 50.1 | 40.1 |  |
| N_MSH_ (Nb α-MSH per mm²) | 2.3E^+12^ | 1.9E^+12^ | (N_COOH_*C_Y_)/100 |
| Density α-MSH (pmol/mm²)^a^ | 3.9 | 3.1 | (N_MSH_/N_A_)*1.0E^+12^ |
| Average α-MSH density (pmol/mm²) | 3.5 ± 0.5 |  |  |

^a^N_A_ is the Avogadro constant

References

1. Takahashi Y, Tadokoro H. Structural Studies of Polyethers, (-(CH2)m-O-)n. X. Crystal Structure of Poly(ethylene oxide). Macromolecules. 1973;6(5):672-5.
