# Supplementary material for: Beneficial Effect of Covalently Grafted α-MSH on Endothelial Release of Inflammatory Mediators for Applications in Implantable Devices
Source: PLoS One. 2016 Mar 3;11(3):e0150706. doi: 10.1371/journal.pone.0150706 (PMC4777356; doi:10.1371/journal.pone.0150706)
Supplement: S3 Text — (DOCX) [file pone.0150706.s003.docx]

A monolayer of thiolated α-MSH was directly deposited onto bare gold (labelled MSH in fig. A), as described in the manuscript. We then quantified the anti-inflammatory properties of this surface using the same protocol described in the materials and methods section. Results of the impact of the surfaces on IL-6 production are shown in Fig. A. Thiolated MSH onto gold does not fare better than other surface types in reducing HUVEC IL-6 production.

Fig. A. HUVEC IL-6 production on the surfaces used in this study. IL-6 production of endothelial cells that were re-plated onto bare gold, EO_7_-COOH, EO_7_-MSH surfaces, a thiolated α-MSH monolayer and culture dish (control) in culture medium containing 1µg/mL lipopolysaccharide (LPS+) or normal culture medium (LPS-). Error bars represent standard deviations. The number of replicates is n=2. Each surface type was prepared in triplicate.
